# Multimodal prehabilitation in colorectal cancer surgery: randomized clinical trial

**DOI:** 10.1093/bjsopen/zrag167

**Published:** 2026-10-23

**Authors:** Felix Bjerregaard, Klas Pekkari, Daniel Asklid, Claes Anderin, Karin Westberg, Richard Marsk, Ulf O Gustafsson

**Affiliations:** Department of Clinical Sciences at Danderyd Hospital, Karolinska Institutet, Stockholm, Sweden; Department of Surgery and Urology, Danderyd Hospital, Stockholm, Sweden; Department of Clinical Sciences at Danderyd Hospital, Karolinska Institutet, Stockholm, Sweden; Department of Surgery, Capio Kirurgkliniken Stockholm AB, Stockholm, Sweden; Department of Clinical Sciences at Danderyd Hospital, Karolinska Institutet, Stockholm, Sweden; Department of Surgery, Ersta Hospital, Stockholm, Sweden; Department of Clinical Sciences at Danderyd Hospital, Karolinska Institutet, Stockholm, Sweden; Department of Surgery, Ersta Hospital, Stockholm, Sweden; Department of Surgery and Urology, Danderyd Hospital, Stockholm, Sweden; Department of Molecular Medicine and Surgery, Karolinska Institutet, Stockholm, Sweden; Department of Clinical Sciences at Danderyd Hospital, Karolinska Institutet, Stockholm, Sweden; Department of Pelvic Cancer, Division of Coloproctology, Center for Digestive Diseases, Karolinska University Hospital, Stockholm, Sweden; Department of Clinical Sciences at Danderyd Hospital, Karolinska Institutet, Stockholm, Sweden; Department of Molecular Medicine and Surgery, Karolinska Institutet, Stockholm, Sweden; Department of Pelvic Cancer, Division of Coloproctology, Center for Digestive Diseases, Karolinska University Hospital, Stockholm, Sweden

**Keywords:** postoperative complications, enhanced recovery after surgery (ERAS), colorectal surgery, functional outcome, prehabilitation

## Abstract

**Background:**

Colorectal cancer (CRC) surgery remains associated with substantial postoperative morbidity despite implementation of enhanced recovery after surgery (ERAS). Prehabilitation improves surgical outcomes, but evidence remains inconsistent. This study evaluated whether multidisciplinary prehabilitation reduces postoperative complications and whether treatment effects vary according to baseline physical function.

**Methods:**

This single-centre randomized clinical trial was conducted at a tertiary referral hospital in Stockholm, Sweden (28 October 2021–23 April 2025). Adults with histologically confirmed CRC scheduled for elective resection were randomized 1 : 1 to multidisciplinary prehabilitation (medication review, nutritional counselling, supervised exercise, and targeted risk optimization) or standard care (ERAS). Analyses followed a modified intention-to-treat (mITT) principle. Prehabilitation was initiated within 3 days of enrolment. The primary outcome of interest was postoperative complications (Clavien–Dindo grade ≥ I). Secondary outcomes included severe complications (Comprehensive Complication Index), reoperations, length of hospital stay (LOS), postoperative weight loss, physical performance and function (6-minute walk test, Timed Up and Go test, and 30-second chair stand test), health-related quality of life (EORTC QLQ-C30), mental health (Hospital Anxiety and Depression Scale), and correlation with the Disability Rating Index (DRI) score.

**Results:**

Among 328 randomized patients, 242 underwent CRC surgery and were included in the mITT analysis (120 control, 122 intervention). Postoperative complications occurred in 46.2% of patients, with no significant difference between the intervention and control groups (47.5% *versus* 45.0%, respectively). Severe complications occurred in 19.0% of patients and were numerically less frequent in the intervention group, but the difference was not statistically significant. LOS and reoperation rates were similar. The intervention group had lower postoperative weight loss and modest improvements in physical performance and patient-reported physical function at 30 days. In exploratory analyses, a significant interaction was observed between treatment allocation and baseline DRI score, indicating greater reduction in severe complications among patients with higher levels of preoperative disability (*P* = 0.011).

**Conclusions:**

Multidisciplinary prehabilitation did not reduce postoperative complications after CRC surgery. However, exploratory analyses suggest that patients with greater preoperative disability derive greater benefit, supporting targeted prehabilitation strategies in higher-risk populations. Registration number: NCT04909567 (https://clinicaltrials.gov/).

## Teaser

Multidisciplinary prehabilitation did not reduce overall postoperative complications after colorectal cancer surgery. However, exploratory analyses suggest that patients with greater preoperative disability derive greater benefit, supporting targeted prehabilitation strategies in higher-risk surgical populations.

## Introduction

Colorectal cancer is one of the most common malignancies worldwide, with approximately 1.2 million new cases annually. In Sweden, the median age at diagnosis is 70 years, and the 5-year survival rate is approximately 60% for men and 65% for women^1^.

Surgical resection with curative intent remains the cornerstone of treatment. The introduction of the enhanced recovery after surgery (ERAS) protocol has improved perioperative care and recovery^2^. Nevertheless, major colorectal cancer surgery, particularly in older patients with multimorbidity, remains associated with substantial postoperative morbidity, prolonged recovery, impaired quality of life, and considerable healthcare utilization^2^.

A growing proportion of patients are older and frail, with reduced physiological reserve, factors that significantly affect surgical tolerance and outcomes^3^.

Prehabilitation has been proposed as an adjunct to the ERAS protocol to improve outcomes; however, studies in colorectal surgery have yielded inconsistent results^4–7^, and routine implementation is not currently recommended^2^. Interpretation is limited by heterogeneity in intervention content and outcome measures, ranging from exercise-only to multimodal programmes including nutritional and psychological support, with varying delivery models and endpoints. Many studies are also underpowered or include selected populations.

Accordingly, adequately powered randomized clinical trials evaluating clinically meaningful outcomes are warranted. This randomized controlled trial aimed to determine whether a structured, supervised, multimodal prehabilitation programme reduces postoperative complications in patients undergoing colorectal cancer surgery.

## Methods

### Study design and setting.

This single-centre open-label randomized clinical trial was conducted at Danderyd Hospital, a tertiary referral hospital in Stockholm, Sweden, serving a catchment population of approximately 700 000 individuals. The Department of Surgery is accredited as an ERAS^®^ Centre of Excellence, and all elective colorectal resections are performed within a standardized ERAS protocol^2^. The study was approved by the Swedish Ethical Review Authority (Dnr 2021-00571). The trial was registered with ClinicalTrials.gov (NCT04909567) on 26 May 2021, where the original protocol is available. The study followed the CONSORT reporting guideline^8^. Patients and the public were not involved in the design, conduct, reporting, or dissemination plans of this study.

Patients referred for suspected colorectal cancer between 28 October 2021 and 23 April 2025 were screened for eligibility. Inclusion criteria were histologically confirmed colorectal adenocarcinoma and planned surgical resection at the study centre. Exclusion criteria included age < 18 years, cognitive impairment precluding informed consent, non-ambulatory status preventing exercise, metastatic disease requiring neoadjuvant treatment, inability to understand Swedish, or planned diverting stoma before enrolment.

Eligible patients provided written informed consent and were randomized 1 : 1 to the intervention or control group using computerized block randomization stratified by tumour location (colon *versus* rectum). Because randomization occurred before completion of the diagnostic work-up and final treatment decisions were made at a multidisciplinary tumour board conference, some patients were excluded after randomization if surgery was not indicated, neoadjuvant treatment was required, or surgery was performed at another institution. Therefore, analyses followed a modified intention-to-treat (mITT) principle (*Fig. 1*).

**Figure 1:**
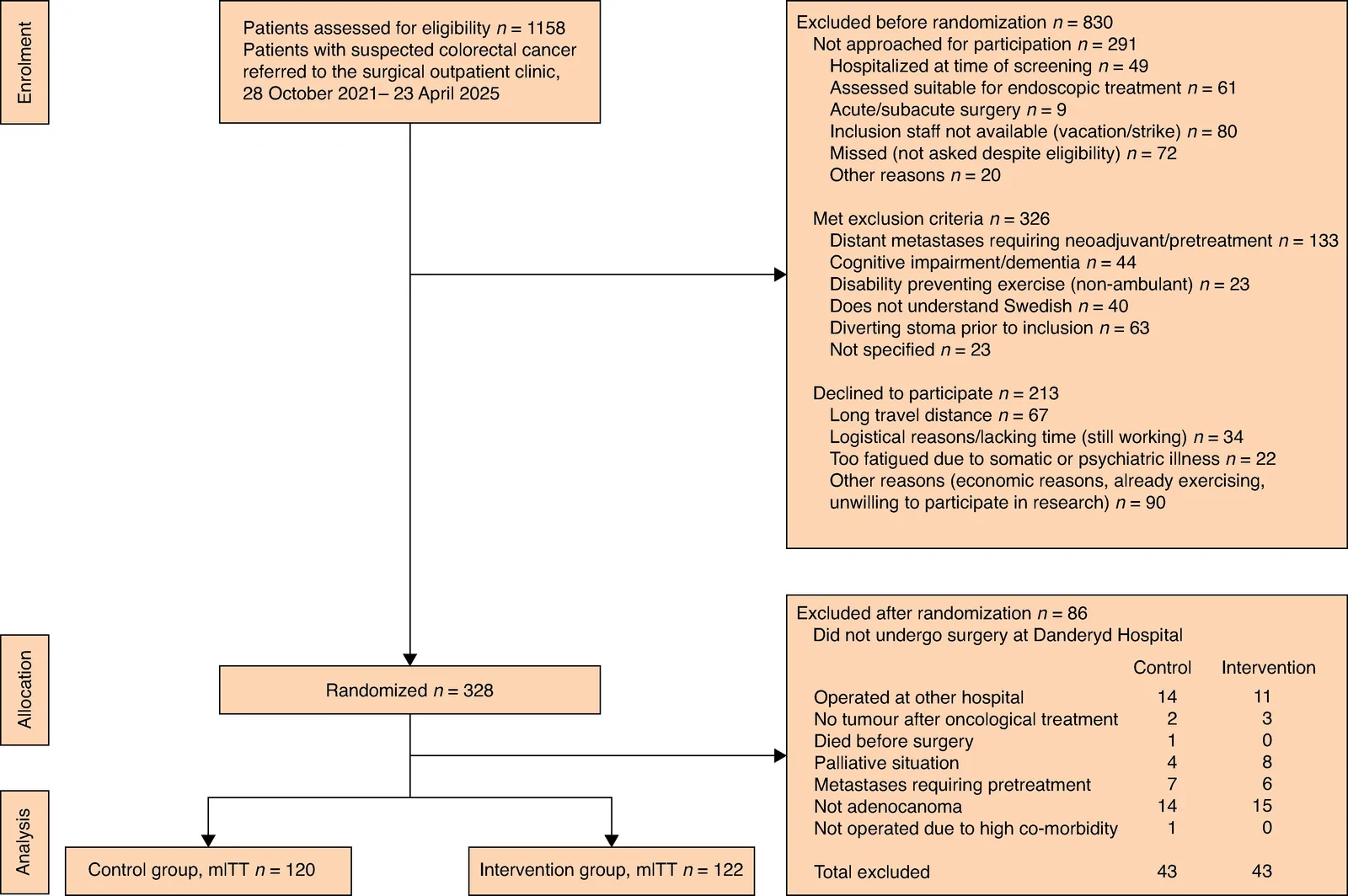
CONSORT flow diagram showing patient screening, randomization, pre- and post-randomization exclusions, and the number of participants included in the modified intention-to-treat (mITT) analysis.

### Assessments

Participants in both groups underwent physical performance testing, including the 6-minute walk test (6MWT)^9^, Timed Up and Go test (TUG)^10^, and the 30-second chair stand test^11^.

Patient-reported outcomes included the Disability Rating Index (DRI)^12^, the EORTC QLQ-C30^13^, and the Anxiety and Depression subscales on the Hospital Anxiety and Depression Scale (HADS-A and HADS-D, respectively)^14^. Nutritional status was assessed using the Patient-Generated Subjective Global Assessment (PG-SGA)^15^.

Laboratory assessments included haemoglobin, ferritin, albumin, liver function tests, creatinine, HbA1c, carbohydrate-deficient transferrin, vitamin D, and carboxyhaemoglobin. Exhaled carbon monoxide was measured to assess smoking status. Assessments were performed at baseline (within 3 days of enrolment), within 7 days before surgery, and 30 days after surgery.

### Perioperative care

All patients underwent surgery within a standardized ERAS pathway comprising 24 predefined perioperative elements^2^. Compliance with ERAS components was prospectively recorded in the ERAS Interactive Audit System database^16^.

### Intervention

Participants in the intervention group started multidisciplinary prehabilitation within 3 days of enrolment. All interventions continued until surgery. The programme included pharmacist-led medication review, individualized nutritional counselling by a dietician, and physiotherapist-supervised exercise training. All participants received initial psychosocial counselling.

Patients were classified as high risk according to predefined criteria including estimated mortality risk > 5% using the American College of Surgeons National Surgical Quality Improvement Program Surgical Risk Calculator^17^, American Society of Anesthesiologists grade ≥ III, an inability to walk at least 400 m during the 6MWT, poorly controlled diabetes, or estimated glomerular filtration rate < 45 ml/min/1.73 m^2^. High-risk patients were reviewed at a multidisciplinary conference including surgeons, anaesthesiologists, and relevant specialists.

Exercise sessions were conducted twice weekly, individually adapted to baseline functional capacity, and targeted moderate-to-high intensity corresponding to a Borg rating of perceived exertion of 14–17^18^. Participants were encouraged to perform additional home-based exercise and maintained training diaries.

Control group participants received standard preoperative care according to the ERAS protocol^2^. Medical conditions identified during outpatient evaluation were referred to appropriate specialists or primary care.

### Outcomes of interest

The primary outcome was postoperative complications, defined as Clavien–Dindo grade ≥ I^19^. This represented a protocol amendment from the originally specified outcome of severe complications (Clavien–Dindo grade ≥ IIIb), implemented approximately 6 months before study completion and before database lock and outcome analyses. Severe complications were analysed as a prespecified secondary outcome.

Secondary surgical outcomes included severe complications, defined as a Comprehensive Complication Index (CCI) score ≥ 26.2^20^, reoperations, surgical complications, length of hospital stay (LOS), and postoperative weight loss. Secondary patient-centred outcomes included changes in physical performance (6MWT, TUG, chair stand), physical function (DRI), health-related quality of life (EORTC QLQ-C30 summary score), and mental health (HADS).

All outcome assessments, including complication registration and performance tests, were performed in a non-blinded manner.

### Statistical analyses

The original sample size calculation was based on severe postoperative complications (Clavien–Dindo grade ≥ IIIb). Following the protocol amendment redefining the primary outcome to Clavien–Dindo grade ≥ I, the sample size was recalculated approximately 6 months before study completion. Assuming a complication rate of 45% and an absolute reduction to 27%, 230 evaluable patients were required for 80% power with a two-sided α of 0.05. Allowing for up to 30% postrandomization exclusions, the target sample size was 328 randomized patients.

All analyses followed the mITT principle. In accordance with contemporary reporting standards, emphasis was placed on the magnitude and precision of treatment effects rather than sole reliance on *P-*values. Effect estimates are presented with their 95% confidence interval (c.i.). All tests were two-sided, and statistical significance was inferred when the c.i. did not include zero.

Binary outcomes were analysed using generalized linear models with a binomial distribution and identity link to estimate absolute risk differences. Continuous outcomes measured at baseline and after surgery were analysed using analysis of covariance, adjusting for baseline values to improve precision. Continuous outcomes without baseline measurements were compared between groups using linear regression; when distributional assumptions were not met, quantile regression was applied.

Missing data for functional outcomes and body weight at 30 days after surgery (*Table S1*) were handled using multiple imputation by chained equations under a missing-at-random assumption. The imputation model included treatment group, prespecified baseline covariates, baseline outcome values, and postoperative morbidity indicators (CCI, reoperation, and LOS). Estimates were combined using Rubin's rules. Surgical outcomes were complete and were not imputed. A sensitivity analysis was performed in which the primary outcome was restricted to predefined postoperative complications, excluding non-specific symptoms, signs, and laboratory abnormalities.

Exploratory post hoc analyses were conducted to assess potential heterogeneity of treatment effects according to baseline functional status. Interaction terms between treatment assignment and baseline DRI and 6MWT were added to binomial regression models. Marginal effects were estimated across the observed range of baseline values. These analyses were considered hypothesis generating and were not adjusted for multiple comparisons.

All statistical analyses were performed using Stata®/BE version 17.0 (StataCorp LLC, College Station, TX, USA).

## Results

Participant flow is shown in *Fig. 1*. Among 1158 patients presenting with suspected colorectal cancer, 867 were assessed for eligibility. After exclusion of 326 patients who met the exclusion criteria and 213 who declined participation, 328 were randomized (163 to the control group and 165 to the intervention group). Because randomization occurred before completion of the diagnostic work-up, 86 patients were excluded after randomization. The final mITT population comprised 242 patients who underwent colorectal cancer surgery at the study centre (120 control, 122 intervention). The median time from inclusion to surgery was 4.8 (interquartile range (i.q.r.) 4–6) weeks.

Baseline characteristics were well balanced between the control and intervention groups (*Table 1*). Overall baseline characteristics for the study population are summarized here in the text; group-specific values are presented in *Table 1*. The median age for the study population was 74 years, and 51.7% of patients were men. Most patients (78.1%) had an American Society of Anesthesiologists grade of I–II. Hypertension and diabetes were the most prevalent co-morbidities. Tumour location was the right colon in 47.1% of patients and the rectum in 29.8% of patients, and 39.7% of patients had stage III disease on preoperative radiological staging (clinical tumour node metastasis (cTNM) staging). Minimally invasive resection was performed in 94.6% of patients, including 35.5% robot-assisted procedures, with a conversion rate of 5.7%.

**Table 1:** Baseline characteristics of the study population

	Intervention group (*n* = 122)	Control group (*n* = 120)
Age (years), median (i.q.r.)	73.0 (63.0–80.0)	74.0 (65.5–81.0)
**Sex**		
Female	58 (47.5%)	59 (49.2%)
Male	64 (52.5%)	61 (50.8%)
BMI (kg/m^2^), median (i.q.r.)	26.1 (23.9–28.7)	25.2 (22.6–29.1)
**ASA grade**		
I	26 (21.5%)	24 (20.0%)
II	68 (56.2%)	71 (59.2%)
III	26 (21.5%)	25 (20.8%)
IV	1 (0.8%)	0 (0.0%)
**NSQIP risk**		
Of 30-day mortality (%), median (i.q.r.)	0.3 (0.1–1.1)	0.5 (0.1–1.2)
Of serious complications (%), median (i.q.r.)	9.3 (6.2–14.0)	11.2 (6.7–14.5)
Hypertension	76 (62.3%)	67 (55.8%)
Heart failure	10 (8.2%)	9 (7.5%)
Previous MI	10 (8.2%)	5 (4.2%)
COPD	7 (5.8%)	5 (4.2%)
Diabetes	20 (16.4%)	23 (19.2%)
Chronic kidney disease	7 (5.7%)	12 (10.0%)
Neurological disorder	3 (2.5%)	2 (1.7%)
CPAP therapy	5 (4.1%)	7 (5.8%)
Previous abdominal surgery	52 (43.3%)	50 (42.0%)
Smoker	9 (7.4%)	4 (3.3%)
No. of standard drinks/week, median (i.q.r.)	2.0 (0.0–6.0)	2.0 (0.0–5.0)
Corticosteroid therapy	3 (2.5%)	2 (1.7%)
NSAID therapy	3 (2.5%)	2 (1.7%)
Anticoagulant therapy	23 (18.9%)	20 (16.7%)
Antiplatelet therapy	17 (13.9%)	14 (11.7%)
PG-SGA B/C	18 (14.8%)	14 (11.7%)
Hb (g/l), median (i.q.r.)	129.5 (112.0–141.0)	126.0 (110.5–137.5)
Albumin (g/l), median (i.q.r.)	36.0 (34.0–39.0)	37.0 (34.0–38.0)
Creatinine (µmol/l), median (i.q.r.)	78.0 (68.0–89.0)	72.5 (62.5–87.0)
HbA1c (mmol/mol), median (i.q.r.)	37.0 (34.0–40.0)	38.0 (35.0–41.0)
6MWD (m), median (i.q.r.)	484.5 (420.0–569.0)	494.0 (406.0–551.0)
30-s Chair Stand Test (*n*), median (i.q.r.)	2.0 (1.0–2.0)	2.0 (1.0–2.0)
TUG (s), median (i.q.r.)	6.0 (6.0–8.0)	7.0 (6.0–8.0)
HRQoL score, median (i.q.r.)	82.7 (71.6–90.3)	86.5 (75.6–92.9)
Physical function (DRI), median (i.q.r.)	17.0 (6.0–33.0)	15.0 (4.0–32.0)
**Mental health**		
HADS-A, median (i.q.r.)	5.0 (2.0–8.0)	4.0 (1.0–7.0)
HADS-D, median (i.q.r.)	2.0 (1.0–5.0)	2.0 (1.0–4.0)
**Clinical tumour stage (cTNM)**		
Stage I	46 (37.7%)	32 (26.7%)
Stage II	27 (22.1%)	28 (23.3%)
Stage III	44 (36.1%)	52 (43.3%)
Stage IV	2 (1.6%)	0 (0.0%)
Unknown	3 (2.5%)	8 (6.7%)
Neoadjuvant chemotherapy	8 (6.6%)	8 (6.7%)
Radiation therapy	5 (4.1%)	10 (8.3%)
**Type of procedure**		
Right hemicolectomy	57 (46.7%)	57 (47.5%)
Left hemicolectomy	13 (10.7%)	7 (5.8%)
Sigmoid resection	17 (13.9%)	13 (10.8%)
Rectal resection	35 (28.7%)	37 (30.8%)
Total/subtotal colectomy	0 (0.0%)	6 (5.0%)
**Surgical approach**		
Laparoscopic surgery	78 (63.9%)	65 (54.2%)
Robot-assisted surgery	40 (32.8%)	46 (38.3%)
Open	4 (3.3%)	9 (7.5%)

Values are *n* (%) unless otherwise stated. No statistical tests were performed. i.q.r., interquartile range; BMI, body mass index; ASA, American Society of Anesthesiologists; NSQIP, National Surgical Quality Improvement Program; MI, myocardial infarction; COPD, chronic obstructive pulmonary disease; CPAP, continuous positive airway pressure; NSAID, non-steroidal anti-inflammatory drug; PG-SGA B/C, Patient-Generated Subjective Global Assessment class B or C, indicating patients at risk of malnutrition or who are malnourished; Hb, haemoglobin; 6MWD, 6-minute walk distance; TUG, Timed Up and Go test; HRQoL, health-related quality of life; DRI, Disability Rating Index; HADS-A, Hospital Anxiety and Depression Scale Anxiety subscale; HADS-D, Hospital Anxiety and Depression Scale Depression subscale; cTNM, clinical tumour node metastasis staging system.

### Intervention adherence

Participants completed a median of 6 (i.q.r. 4–8) supervised exercise sessions and 7 (i.q.r. 7–12) unsupervised sessions. Nutritional counselling by a dietician was provided to 116 patients (95.1%), and 97 patients (79.5%) adhered to at least 75% of the recommended nutritional measures. Among patients classified as PG-SGA class B or C, 73% reported taking oral nutritional supplements as prescribed. Medication review by a pharmacist was conducted in 114 patients (93.4%), and 39 patients (32.0%) were identified as high risk and discussed at the multidisciplinary high-risk conference. Initial psychosocial counselling was completed in 108 patients (88.5%).

### Compliance with the ERAS protocol

Mean compliance was similar between the intervention and control groups with regard to preoperative (7 interventions; 84.2% *versus* 84.5%, respectively) and intraoperative (11 interventions; 85.1% *versus* 85.2%, respectively) ERAS measures. Postoperative compliance (6 interventions) was also comparable between the two groups.

### Primary outcome

Overall, 112 patients (46.2%) experienced at least one postoperative complication. The incidence of complications was 47.5% (58 of 122) in the intervention group and 45.0% (54 of 120) in the control group (*Table 2*), corresponding to an absolute risk difference of 2.5 percentage points, which was not statistically significant.

**Table 2:** Surgical outcomes

	Intervention (*n* = 122)	Control (*n* = 120)	Absolute risk difference*
**Complications**			
Any	58 (47.5%)	54 (45.0%)	2.5 (−10.0, 15.1)
Severe: CCI ≥ 26.2	18 (14.8%)	28 (23.3%)	−8.6 (−18.4, 1.3)
Severe: CD ≥ IIIb	15 (12.3%)	21 (17.5%)	−5.2 (−14.1, 3.7)
Reoperations	13 (10.7%)	20 (16.7%)	−6.0 (−14.7, 2.6)
Surgical complications†	29 (23.8%)	32 (26.7%)	−2.9 (−13.9, 8.1)
Medical complications	6 (4.9%)	12 (10.0%)	−5.1 (−11.7, 1.5)
Infectious complications	33 (27.1%)	21 (17.5%)	9.5 (−0.9, 20.0)
**Most common complications**			
Postoperative ileus	16 (13.1%)	15 (12.5%)	1.4 (−6.9, 9.8)
Urinary tract infection	14 (11.5%)	11 (9.2%)	2.3 (−5.4, 10.0)
Anastomotic leakage	8 (6.6%)	16 (13.3%)	−6.8 (−14.3, 0.7)
Fever/elevated CRP‡	12 (9.8%)	10 (8.3%)	1.5 (−5.7, 8.8)
Mortality	0 (0.0%)	1 (0.8%)	NE

Values are *n* (%) unless otherwise stated. *Absolute risk difference between the intervention and control groups (in percentage points) with 95% confidence intervals in parentheses. †Surgical complications were defined as adverse events directly related to the surgical intervention and surgical trauma. ‡Fever or elevated CRP of unknown origin requiring antibiotic treatment. CCI, Comprehensive Complication Index; CD, Clavien–Dindo; CRP, C-reactive protein; NE, not estimated due to too few events.

### Secondary outcomes

Secondary outcomes are presented in *Table 2*, *Table 3*, and *Fig. 2*. Severe postoperative complications (CCI ≥ 26.2) occurred in 46 patients (19.0%), with no statistically significant difference between the intervention and control groups. Surgical complications were the most common type, followed by infectious and medical complications; none of these outcomes differed significantly between groups.

**Figure 2:**
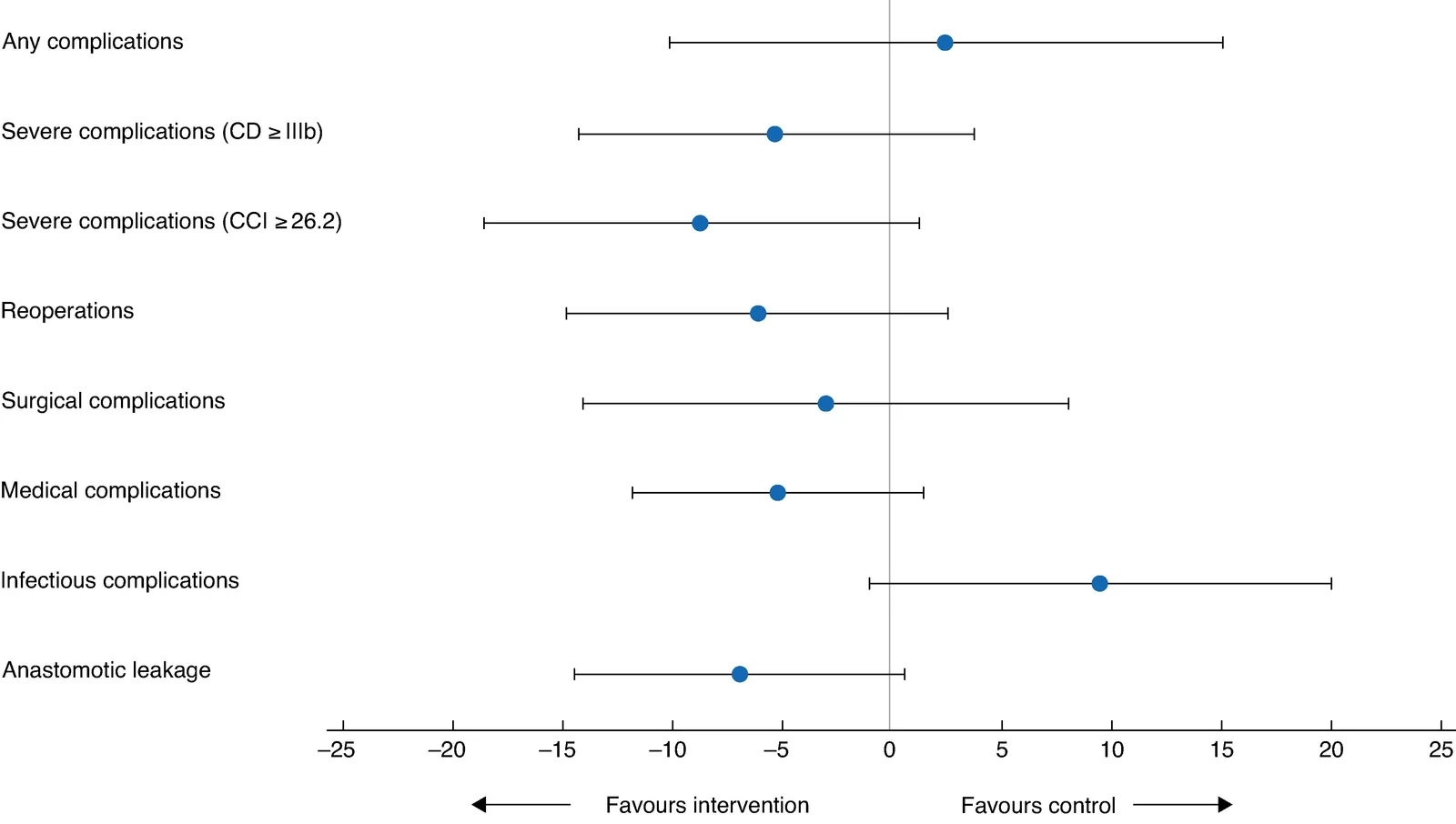
Forest plot showing absolute risk differences with 95% confidence intervals for postoperative complications and reoperations Negative values favour the intervention group and positive values favour the control group. CD, Clavien–Dindo; CCI, Comprehensive Complication Index.

**Table 3:** Postoperative recovery items at the 30-days follow-up

	Intervention	Control	Adjusted mean difference*
**Nutritional status**			
Weight (kg), median (i.q.r.)	78 (69–85)	73.7 (63–85)	1.0 (0.1, 2.0)
PG-SGA B/C	16 (13.1%)	24 (20.0%)	−8.5 (−18.5, 1.4)†
**Functional status**			
6MWD (m), median (i.q.r.)	477 (400–559)	475.5 (378–537)	22 (−4, 48)
30-s Chair Stand Test (*n*), median (i.q.r.)	12 (10–16)	11.5 (8–15)	1.2 (0.0, 2.4)
TUG (s), median (i.q.r.)	7 (5–8)	7 (6–9)	−0.7 (−1.5, 0.1)
Physical function (DRI), median (i.q.r.)	24 (12–42)	28 (12.5–43)	−4.8 (−9.7, −0.0)
**Mental well being**			
HRQoL‡ score, median (i.q.r.)	82.5 (69.2–89.9)	83.8 (68.8–92.0)	2.0 (−1.6, 6.0)
Mental health			
HADS-A score, median (i.q.r.)	2 (0–6)	2 (0–5)	−0.0 (−0.8, 0.8)
HADS-D score, median (i.q.r.)	1.5 (1–4)	1 (0–5)	−0.4 (−1.1, 0.3)

Values are *n* (%) unless otherwise stated. *Adjusted mean difference using analysis of covariance adjusting for baseline values between the intervention and control groups, except †absolute risk difference (in percentage points) between the intervention and control groups; values in parentheses are 95% confidence intervals. ‡HRQoL according to the EORTC QLQ-C30 summary score (range 0–100), with higher scores indicating better HRQoL. i.q.r., interquartile range; PG-SGA B/C, Patient-Generated Subjective Global Assessment class B or C, indicating patients at risk of malnutrition or who are malnourished; 6MWD, 6-minute walk distance; TUG, Timed Up and Go test; DRI, Disability Rating Index; HRQoL, health-related quality of life; HADS-A, Hospital Anxiety and Depression Scale Anxiety subscale; HADS-D, Hospital Anxiety and Depression Scale Depression subscale.

The most frequent complications were ileus, urinary tract infection, anastomotic leakage, and fever or elevated C-reactive protein requiring antibiotic therapy. A detailed distribution of all complications is presented in *Table S2*. Anastomotic leakage occurred less frequently in the intervention group, although the between-group difference was not statistically significant. Reoperation was required in 33 patients, with no statistically significant difference between groups.

The median LOS was 3 days and did not differ between groups. The mean LOS was 1.2 days shorter in the intervention group (95% c.i. −2.7 to 0.2), although this difference was not statistically significant. The distribution was right skewed, with prolonged hospitalizations (> 3 weeks) observed only in the control group.

The mean postoperative weight loss in the overall study population was 2.4 kg. Weight loss was lower in the intervention group, with a between-group difference of 1.0 kg (*Table 3*). Consistent with this finding, the risk of being classified as PG-SGA class B or C at the 30-day follow-up was 8.5 percentage points lower in the intervention group after adjustment for baseline values, although the difference was not statistically significant.

Functional recovery and patient-reported outcomes at 30 days are presented in *Table 3*. Between-group differences represent changes from baseline and were estimated using models adjusted for the corresponding baseline value of each outcome.

After adjustment, the intervention group walked 22 m further on the 6MWT, completed the TUG 0.7 s faster, and performed 1.2 additional repetitions on the 30-second Chair Stand Test compared with the control group.

The DRI score was 4.8 points lower in the intervention group, indicating better physical function. The EORTC QLQ-C30 summary score was 2.0 points higher in the intervention group. There was no between-group difference in the HADS-A, and the difference in the HADS-D was −0.4 points.

### Exploratory interaction analyses

Because the intervention was not associated with a difference in overall complications but suggested a reduction in severe complications, exploratory analyses examined potential effect modification by baseline physical function. For severe complications, a significant interaction was observed between treatment allocation and baseline DRI score, indicating greater absolute risk reduction in patients with higher levels of preoperative disability (*P*_interaction_ = 0.011; *Fig. 3*). No interaction was observed according to baseline 6MWT performance (*P*_interaction_ = 0.116). Model calibration showed good agreement between predicted and observed outcomes.

**Figure 3:**
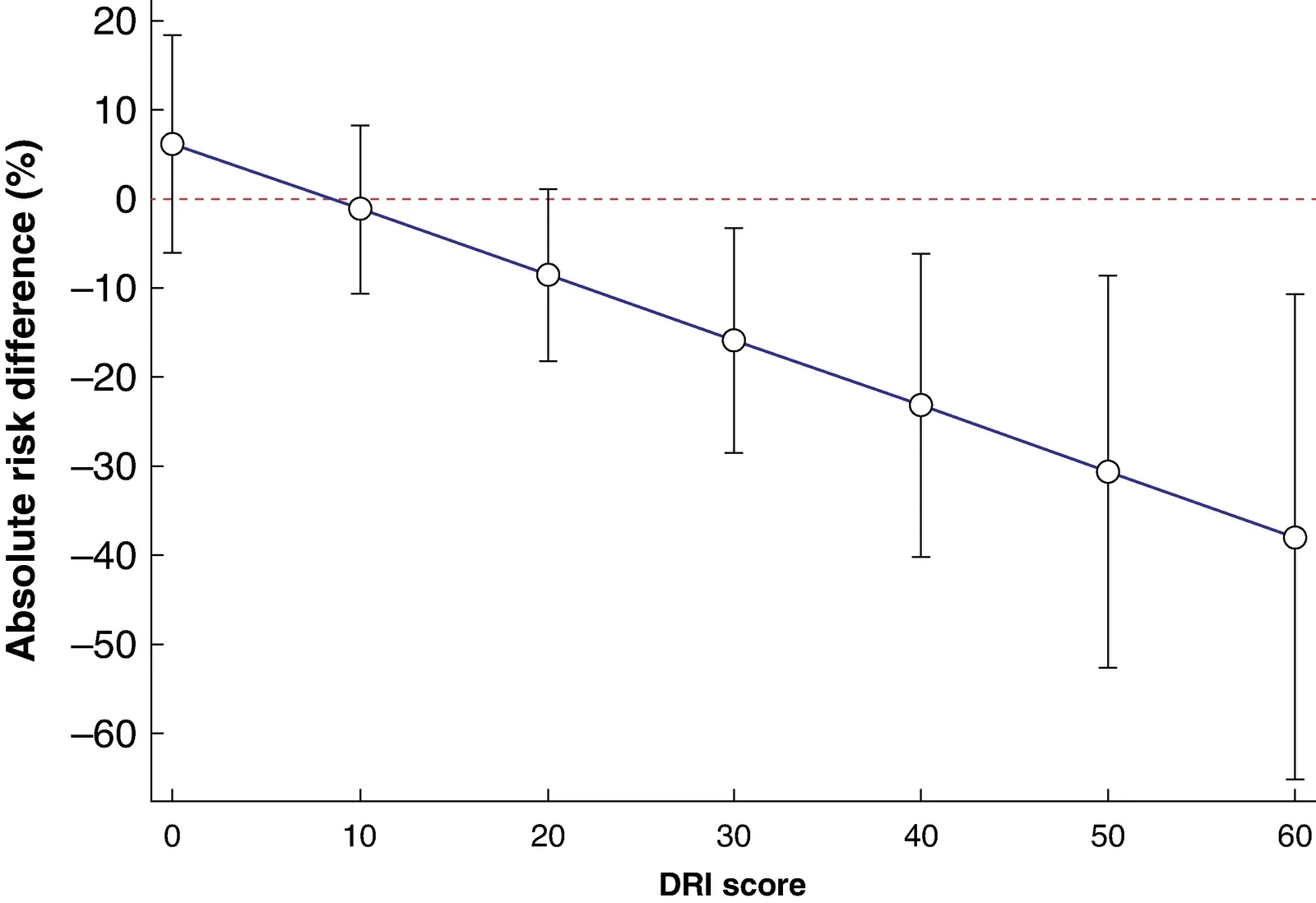
Estimated absolute risk difference in severe postoperative complications according to the baseline DRI score Error bars indicate 95% confidence intervals. The benefit of prehabilitation increases with higher levels of preoperative disability. DRI, Disability Rating Index.

### Sensitivity analyses

A sensitivity analysis restricted to predefined postoperative complications, excluding non-specific symptoms, signs, and laboratory abnormalities, yielded results consistent with the primary analysis (*Tables S3* and *S4*).

## Discussion

This randomized clinical trial, conducted within an ERAS pathway, represents the largest trial of multidisciplinary prehabilitation in colorectal cancer surgery performed under a strict standardized ERAS protocol to date. The primary endpoint was overall postoperative complications, for which no difference between groups was observed and no numerical trend favoured the intervention. However, a consistent numerical trend towards fewer severe complications was observed in the intervention group, although the difference did not reach statistical significance. This pattern appeared to be driven by a lower incidence of major surgical events requiring reoperation, such as anastomotic leakage. Similar patterns were observed for LOS and measures of functional recovery, whereas no differences were detected in patient-reported quality of life or mental health.

Together, these findings suggest that multidisciplinary prehabilitation may have limited impact on overall postoperative morbidity in an unselected ERAS population. However, exploratory analyses indicated that patients with greater preoperative disability derived greater benefit from the intervention, supporting a strategy of targeting multidisciplinary prehabilitation to higher-risk surgical populations.

Previous randomized trials of multidisciplinary prehabilitation have been heterogeneous in design, population, and intervention content^21–25^. Programmes have ranged from exercise-only approaches to multimodal interventions including nutritional optimization and medical risk assessment, and outcome definitions have varied substantially. Many studies have also relied on unsupervised interventions or focused primarily on functional outcomes rather than clinically relevant surgical endpoints.

Following the multicentre PREHAB trial published in 2023^5^, the present study represents the second largest randomized trial and the largest single-centre evaluation of multidisciplinary prehabilitation in colorectal cancer surgery. Importantly, the present trial was conducted within a strictly implemented ERAS pathway with prospectively recorded compliance using the ERAS Interactive Audit System, allowing detailed monitoring of adherence to perioperative ERAS elements and ensuring that all patients were treated within a highly standardized surgical environment. This level of protocol fidelity likely differs from that of many multicentre trials and may partly explain differences in observed treatment effects.

In the PREHAB trial^5^, severe complications were defined as a CCI score of ≥ 20, and the intervention reduced the rate from 29.7% to 17.1%, corresponding to an absolute risk reduction of 12.6 percentage points. In the present study, a stricter threshold (CCI ≥ 26.2), corresponding to at least one Clavien–Dindo grade IIIa complication, was applied. Using this definition, the observed reduction in severe complications was 8.6 percentage points, from 23.3% in the control group to 14.8% in the intervention group. Although smaller, as expected with a stricter definition of severity, the effect size was broadly consistent with the findings of the PREHAB trial^5^.

Although overall complication rates were unchanged, the intervention appeared to attenuate complication severity, suggesting that multidisciplinary prehabilitation may enhance resilience to surgical stress rather than prevent complications from occurring. Exploratory analyses indicated that this effect was concentrated among patients with greater preoperative disability. A significant interaction was observed for baseline DRI, with progressively larger treatment effects at higher disability levels, whereas no interaction was observed for baseline walking capacity measured using the 6MWT.

Although multidisciplinary prehabilitation was not associated with a reduction in overall postoperative complications in this unselected colorectal cancer population treated within a mature ERAS pathway, exploratory analyses suggested greater benefit among patients with higher levels of preoperative disability. This finding is biologically plausible and consistent with previous studies in selected higher-risk populations^26,27^. However, because the analysis was exploratory and the primary endpoint was not met, the results should be considered hypothesis generating and require confirmation in future studies.

The study was underpowered to detect statistically significant differences in severe complications. Recruitment was more challenging than anticipated, partly reflecting the large catchment area of the study centre, and some eligible patients were not approached because of logistical constraints during routine clinical care. The proportion of patients meeting exclusion criteria was similar in the eligible population and among those randomized, suggesting a limited risk of major selection bias. However, the moderate inclusion rate highlights an important challenge for the implementation of multidisciplinary prehabilitation in routine clinical practice. Many patients were ineligible because of diagnostic uncertainty, treatment pathway changes, neoadjuvant therapy, logistical constraints, or inability to participate in a supervised programme. Although potentially beneficial, supervised multidisciplinary prehabilitation may be difficult to implement on a broad scale, and more flexible delivery models may be needed to increase accessibility and uptake.

Additional study limitations should be acknowledged. First, the intervention consisted of multimodal multidisciplinary prehabilitation, making it difficult to determine which individual components contributed most to the observed effects. Second, the duration of the multidisciplinary prehabilitation programme varied depending on the interval between enrolment and surgery, which may have influenced the magnitude of the intervention effect. Third, all patients were treated within a strictly implemented ERAS pathway. Although this strengthens standardization of perioperative care, it may also have attenuated differences between groups because both groups received optimized perioperative management. Finally, a substantial proportion of eligible patients declined participation. Because no data were collected from non-participants, potential differences between participants and non-participants could not be assessed. Consequently, selection bias and reduced generalizability cannot be excluded.

This study also has several strengths. The single-centre design ensured uniform perioperative management and consistent delivery of the intervention, while ERAS compliance was prospectively recorded using the validated ERAS Interactive Audit System. This level of protocol fidelity strengthens internal validity. In addition, data completeness for surgical outcomes was high, and the pragmatic conduct of the trial enhances the relevance of the findings for routine clinical practice.

In conclusion, multidisciplinary prehabilitation did not reduce overall postoperative complications after colorectal cancer surgery within a standardized ERAS pathway. However, exploratory analyses suggest that patients with greater preoperative disability derive greater benefit, supporting a strategy of targeted multidisciplinary prehabilitation in higher-risk surgical populations. Future studies should evaluate risk-stratified approaches to maximize the effectiveness of multidisciplinary prehabilitation.

## Supplementary Material

zrag167_Supplementary_Data

## Data Availability

Due to Swedish legal restrictions and the current ethics approval for the study, the data will not be publicly shared, but the research group can provide descriptive data in table form.
