# Supplementary material for: Multimodal prehabilitation in colorectal cancer surgery: randomized clinical trial
Source: BJS Open. 2026 Oct 23;10(5):zrag167. doi: 10.1093/bjsopen/zrag167 (PMC13692687; doi:10.1093/bjsopen/zrag167)
Supplement: Supplementary file 1 [file zrag167_supplementary_data.docx]

**Multimodal Prehabilitation in Colorectal Cancer Surgery - A Randomised Clinical Trial**

Felix Bjerregaard MD^1,2^, Klas Pekkari MD, PhD^1,3^, Daniel Asklid MD, PhD^1,4^, Claes Anderin MD, PhD^1,4^, Karin Westberg MD, PhD^2,5^, Richard Marsk MD, PhD^1,6^, Ulf O Gustafsson MD, PhD^1,5,6^

^1^Department of Clinical Sciences at Danderyd hospital, Karolinska Institutet, Stockholm, Sweden

^2^Department of Surgery and Urology, Danderyd Hospital, Stockholm, Sweden

^3^Department of Surgery, Capio Kirurgkliniken Stockholm AB, Stockholm, Sweden

^4^Department of Surgery, Ersta Hospital, Stockholm, Sweden

^5^ Department of Molecular Medicine and Surgery, Karolinska Institutet, Stockholm, Sweden

^6^ Department of Pelvic Cancer, Division of Coloproctology, Center for Digestive Diseases, Karolinska University Hospital

**Corresponding author.** Felix Bjerregaard, MD, Consultant Surgeon, Department of Surgery, Danderyds Hospital & Department of Clinical Sciences, Danderyds Hospital, Karolinska Institutet. 18288, Danderyd, Stockholm, Sweden. Tel: +46737035793; e-mail: [felix.bjerregard@regionstockholm.se](mailto:felix.bjerregard@regionstockholm.se)

**Supplementary Materials - Index**

| **Supplementary Tables** |  |
| --- | --- |
| Table S1; Variables with missing data | *page 2* |
| Table S2; Detailed distribution of postoperative complications by treatment group.  Table S3: Detailed distribution of postoperative complications registered in the ERAS databas  Table S4: Surgical outcomes in a sensitivity analysis restricted to predefined postoperative complications registered in the ERAS database | *page 3*  *page 5*  *page 6* |

**Supplementary table S1. Variables with missing data.**

| variable | missing (n) | missing (%) |
| --- | --- | --- |
|  |  |  |
| DRI (1) | 10 | 4.1 |
| DRI (2) | 44 | 18.2 |
| HAD-A (1) | 13 | 5.4 |
| HAD-A (2) | 24 | 9.9 |
| HAD-D (1) | 12 | 5.0 |
| HAD-D (2) | 22 | 9.1 |
| TUG (1) | 10 | 4.1 |
| TUG (2) | 24 | 9.9 |
| Chair stand test (1) | 17 | 7.0 |
| Chair stand test (2) | 21 | 8.7 |
| 6-MWT (1) | 10 | 4.1 |
| 6-MWT (2) | 21 | 8.7 |
| EORTC QLQ-C30 summary score (1) | 9 | 3.7 |
| EORTC QLQ-C30 summary score (2) | 21 | 8.7 |
| weight (2) | 19 | 7.9 |
| albumin (1) | 1 | 0.4 |
| albumin (2) | 16 | 6.6 |

(1) *Indicates data at baseline*
(2) *Indicates data at 30 days follow-up*

| **Supplementary table S2. Detailed distribution of postoperative complications by treatment group.** | | | |
| --- | --- | --- | --- |
|  |  |  |  |
|  | **Control** | **Intervention** | **Total** |
|  | |  |  |
| **Surgical complications** | |  |  |
| Wound or port-site dehiscence | 3 | 3 | 6 |
| Anastomotic leakage | 16 | 8 | 24 |
| Bowel ischemia | 1 | 0 | 1 |
| Gastrointestinal bleeding | 1 | 2 | 3 |
| Ileus (paralytic ileus) | 15 | 16 | 31 |
| Intra-abdominal abscess | 7 | 3 | 10 |
| Wound bleeding | 1 | 0 | 1 |
| Subcutaneous emphysema | 0 | 1 | 1 |
| Parastomal hernia | 1 | 0 | 1 |
|  |  |  |  |
| Total | 45 | 33 | 78 |
|  |  |  |  |
| **Medical complications** | |  |  |
| Hypotension | 1 | 1 | 2 |
| Pulmonary embolism | 2 | 0 | 2 |
| Thrombophlebitis | 0 | 2 | 2 |
| Portal vein thrombosis | 1 | 0 | 1 |
| Dyspnoea | 1 | 0 | 1 |
| Respiratory failure | 2 | 0 | 2 |
| Atrial fibrillation | 1 | 1 | 2 |
| Postoperative anaemia | 2 | 0 | 2 |
| Acute kidney injury | 6 | 2 | 8 |
| Splenic infarction | 0 | 1 | 1 |
| Airway oedema | 1 | 0 | 1 |
|  |  |  |  |
| Total | 17 | 7 | 24 |
|  |  |  |  |
| **Infectious complications** | |  |  |
| Fever/elevated C-reactive protein* | 10 | 12 | 22 |
| Urinary tract infection | 11 | 14 | 25 |
| COVID-19 infection | 1 | 0 | 1 |
| Diverticulitis | 0 | 2 | 2 |
| Surgical site infection | 0 | 5 | 5 |
| Pneumonia | 1 | 3 | 4 |
|  |  |  |  |
| Total | 23 | 36 | 59 |
|  |  |  |  |
| **Other complications** | |  |  |
| Vomiting | 2 | 2 | 4 |
| Confusion (delirium) | 1 | 1 | 2 |
| Severe postoperative pain | 0 | 3 | 3 |
| Urinary retention | 6 | 13 | 19 |
| High-stoma output | 3 | 0 | 3 |
| Haematuria | 0 | 1 | 1 |
| Gastric ulcer | 2 | 0 | 2 |
|  |  |  |  |
| Total | 14 | 20 | 34 |

**Numbers of complications within each group. A single patient may contribute multiple complications.**

*** Fever or elevated C-reactive protein without an identified source that prompted antibiotic treatment.**

**Surgical complications were defined as adverse events directly related to the surgical intervention and surgical trauma.**

| **Supplementary table S3. Detailed distribution of postoperative complications registered in the ERAS database** | | | |
| --- | --- | --- | --- |
|  |  |  |  |
| Type of complication | Intervention | Control | Total |
|  |  |  |  |
| Wound or port-site dehiscence | 3 | 3 | 6 |
| Anastomotic leakage | 8 | 16 | 24 |
| Bowel ischemia | 0 | 1 | 1 |
| Gastrointestinal bleeding | 2 | 1 | 3 |
| Ileus (paralytic ileus) | 16 | 15 | 31 |
| Intra-abdominal abscess | 3 | 7 | 10 |
| Wound bleeding | 0 | 1 | 1 |
| Subcutaneous emphysema | 1 | 0 | 1 |
| Parastomal hernia | 0 | 1 | 1 |
| Suspected intraabdominal infection requiring reoperation | 1 | 0 | 1 |
|  |  |  |  |
| Pulmonary embolism | 0 | 2 | 2 |
| Portal vein thrombosis | 0 | 1 | 1 |
| Dyspnea | 0 | 1 | 1 |
| Respiratory failure | 0 | 2 | 2 |
| Atrial fibrillation | 1 | 1 | 2 |
| Postoperative anemia | 0 | 2 | 2 |
| Acute kidney injury | 2 | 6 | 8 |
| Splenic infarction | 1 | 0 | 1 |
| Airway edema | 0 | 1 | 1 |
|  |  |  |  |
| Urinary tract infection | 14 | 11 | 25 |
| Diverticulitis | 2 | 0 | 2 |
| Surgical site infection | 5 | 0 | 5 |
| Pneumonia | 3 | 1 | 4 |
|  |  |  |  |
| Confusion (delirium) | 0 | 1 | 1 |
| Gastric ulcer | 0 | 2 | 2 |
|  |  |  |  |
| Numbers of complications within each group. A single patient may contribute multiple complications. | | | |

| **Table S4. Surgical outcomes in a sensitivity analysis restricted to predefined postoperative complications registered in the ERAS database** | | | |
| --- | --- | --- | --- |
|  | Intervention (n=122) | Control (n=120) | Absolute risk difference* |
|  |  |  |  |
| Complications, any | 43 (35.3%) | 45 (37.5%) | -2.3 (-14.3 to 9.9) |
| Complications, severe (CCI ≥ 26.2) | 16 (13.1%) | 27 (22.5%) | -9.4 (-19.0 to 2.1) |
| Complications, severe (≥ CD3b) | 14 (11.5%) | 21 (17.5%) | -6.0 (-14.9 to 2.8) |
|  |  |  |  |
| Values are presented as number (%).  *Absolute risk difference between intervention and control, in percentage points with 95% confidence interval.  CCI - Comprehensive Complication Index.  CD3b – Clavien-Dindo classification 3b. | | | |
